# Structural Capture of η^1^-OSO to η^2^-(OS)O Coordination Isomerism in a New Ruthenium-Based SO_2_-Linkage Photoisomer That Exhibits Single-Crystal Optical Actuation

**DOI:** 10.1021/acs.jpcc.2c00170

**Published:** 2022-03-29

**Authors:** Jacqueline M. Cole, David J. Gosztola, Jose de J. Velazquez-Garcia

**Affiliations:** † Cavendish Laboratory, Department of Physics, University of Cambridge, J. J. Thomson Avenue, Cambridge CB3 0HE, U.K.; ‡ ISIS Neutron and Muon Source, STFC Rutherford Appleton Laboratory, Harwell Science and Innovation Campus, Didcot OX11 0QX, U.K.; § Center for Nanoscale Materials, Argonne National Laboratory, 9700 S Cass Avenue, Lemont, Illinois 60439, United States

## Abstract

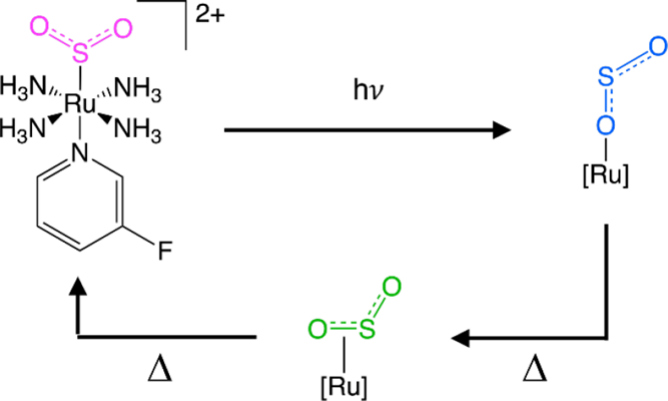

Recent discoveries of a range of single-crystal optical actuators are feeding a new form of materials chemistry, given their broad range of potential applications, from light-induced molecular motors to light sensors and optical-memory media. A series of ruthenium-based coordination complexes that exhibit sulfur dioxide linkage photoisomerization is of particular interest because they exhibit single-crystal optical actuation via either optical switching or nano-optomechanical transduction processes. We report the discovery of a new complex in this series of chemicals, [Ru(SO_2_)(NH_3_)_4_(3-fluoropyridine)]tosylate_2_ (**1**), which forms an η^1^-OSO photoisomer with 70% photoconversion upon the application of 505 nm light. The uncoordinated oxygen atom in this η^1^-OSO photoisomer impinges on one of the arene rings in a neighboring tosylate counter ion of **1** just enough that incipient nano-optomechanical transduction is observed. The structure and optical properties of this actuator are characterized via *in situ* light-induced single-crystal X-ray diffraction (photocrystallography), single-crystal optical absorption spectroscopy and microscopy, as well as single-crystal Raman spectroscopy. These materials-characterization methods were also used to track thermally induced reverse isomerization processes in **1**. One of these processes involves an η^1^-OSO to η^2^-(OS)O transition, which was found to proceed sufficiently slowly at 110 K that its structural mechanism could be determined via a time sequence of photocrystallography experiments. The resulting data allowed us to structurally capture the transition, which was shown to occur via a form of coordination isomerism. Our newfound knowledge about this structural mechanism will aid the molecular design of new [RuSO_2_] complexes with functional applications.

## 1 Introduction

Single-crystal optical actuation is an emerging field of materials chemistry, given the increasing number of optical switches and molecular transducers that have been discovered recently,^1−5^ and their wide-ranging prospective applications: from light-driven molecular rotors,^6^ read-write optical-memory media,^7^ photocatalysis,^8^ to futuristic circuitry for quantum computers.^9^ The functional origin of such actuators lies in the light-induced changes of their molecular structures. To this end, photoisomerization is commonly the molecular mechanism behind the operation of single-crystal optical actuators.^1−3,10−12^

Coordination complexes that exhibit linkage photoisomerization are of particular interest in the photonics domain for several reasons. First, their metal core provides them with good thermal stability. Second, such complexes that form crystalline optical switches are particularly attractive for solid-state memory devices as light-sensitive ligand switches from its dark-state configuration (0) to a photoisomeric structure upon light activation (1) to afford binary encoding in the crystal. Third, a crystal is a pure form of solid-state media; as such, it can be incorporated into a solid-state device with minimal fabrication or processing efforts.

Given their single-crystal form, a good range of coordination complexes that exhibit linkage photoisomerization has been characterized by *in situ* light-induced single-crystal X-ray diffraction,^13−46^ a technique that has been developed over the last few decades and has become known as photocrystallography.^47−51^ “Seeing is believing” with these light-induced crystal structures.

Most of these coordination complexes exhibit optical switching, while a handful of them also display nano-optomechanical transduction:^37−40,43−46^ this is a light-driven switching process in which the photostimulated molecular fragment induces mechanical motion in a neighboring molecule or ion. This handful of coordination complexes all belong to a ruthenium-sulfur-dioxide-based (hereafter [RuSO_2_]) family of materials that have the generic formula, *trans*-[Ru(SO_2_)(NH_3_)_4_X]^*m*+^Y_*n*_, whose ligand, X, lies *trans* to the SO_2_ ligand, and Y is a counter ion; *m* and *n* are integers that relate to charge-balancing requirements, depending on the nature of X and Y.^32−47^ The SO_2_ ligand engages in linkage photoisomerization upon the application of visible light, whereby its S-bound η^1^-SO_2_ dark-state configuration photoisomerizes into one or both of its O-bound η^1^-OSO or side-bound η^2^-(OS)O photoisomers. Wheresoever the O-bound η^1^-OSO photoisomer forms, its uncoordinated (free) oxygen protrudes from the Ru-based cation. The formation of this free oxygen may in turn stimulate nano-optomechanical transduction, depending on its level of proximity to a neighboring anion and the chemical nature of that anion. Thereby, the mechanical motion associated with this transduction process has been witnessed in the form of arene-ring rotation within tosylate or chlorobenzenesulfonate ions of certain [RuSO_2_] complexes.

**Scheme 1:**
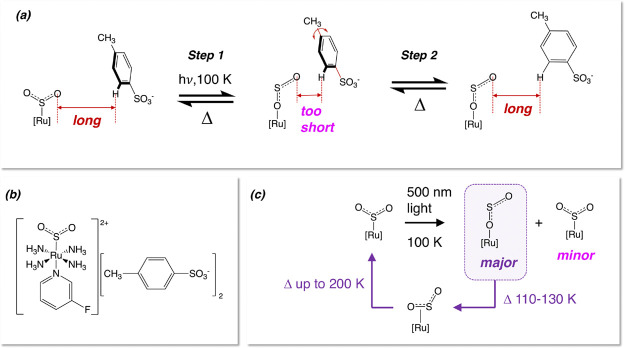
(a) Operational Mechanism of Nano-Optomechanical Transduction for [RuSO_2_] Complexes Proposed by Sylvester et al;^38^ (b) Chemical Schematic of **1** and (c) Its Optical Switching Process

The first [RuSO_2_]-based nano-optomechanical transducers to be discovered were *trans*-[Ru(SO_2_)(NH_3_)_4_(3-chloropyridine)]Y_2_, where Y = tosylate or chlorobenzenesulfonate.^38^ Thereby, Sylvester et al. showed that their dark-state η^1^-SO_2_ ligand could photoconvert to 36.2(4) and 22.4(4)% of a η^1^-OSO photoisomer, the free oxygen of which is so close to the arene ring in one of the anions of the crystallographic asymmetric unit that it rotates in order to alleviate crystal-lattice strain (Scheme 1a). Thus, a small structural change (SO_2_ photoisomerization in the cation) photostimulates a much larger mechanical motion (the arene ring in the anion).

This finding^34^ led to the subsequent discovery of several more [RuSO_2_] complexes that display nano-optomechanical transduction,^39,40,43−46^ including *trans*-[Ru(SO_2_)(NH_3_)_4_(3-bromopyridine)]tosylate_2_ and *trans*-[Ru(SO_2_)(NH_3_)_4_(3-iodopyridine)]tosylate_2_. Both of these complexes were found to exhibit nano-optomechanical transduction with 100% η^1^-OSO photoisomer formation.^44,46^ This 100% SO_2_ photoconversion is important because it opens up real-world opportunities for these materials in solid-state device technologies, where clean photoswitching is a prerequisite for many photonic applications; only fractional SO_2_ photoconversion levels have been realized in all other [RuSO_2_] complexes, as well as many other linkage photoisomers in other types of compounds that have been reported to date.

As well as being the first [RuSO_2_] complexes to show 100% SO_2_ photoconversion, results on *trans*-[Ru(SO_2_)(NH_3_)_4_(3-bromopyridine)]tosylate_2_^44^ and *trans*-[Ru(SO_2_)(NH_3_)_4_(3-iodopyridine)]tosylate_2_^46^ are significant because they complement those of [Ru(SO_2_)(NH_3_)_4_(3-chloropyridine)]tosylate_2_^38^ to form a series of photocrystallographic results on [Ru(SO_2_)(NH_3_)_4_(3-halopyridine)]tosylate_2_ complexes. In turn, these results naturally beg the question as to how the remaining complex in this halogen series behaves.

Accordingly, this study reports the discovery of [Ru(SO_2_)(NH_3_)_4_(3-fluoropyridine)]tosylate_2_ (**1**) (Scheme 1b). We present its synthesis and material characterization that takes the form of photocrystallography, single-crystal Raman spectroscopy, single-crystal optical-absorption spectroscopy, and microscopy. We determine the dark- and light-induced crystal structures of **1** at 100 K, which reveal that one of its tosylate ions is incipient on rotating, such that it is best described as an incipient nano-optomechanical transducer which is induced by the η^1^-SO_2_ to η^1^-OSO photoisomerization process that is depicted in Scheme 1c. We observe progressive changes in the single-crystal optical-absorption profiles of **1** as a function of light-exposure time at 100 K, the nature of which is also a characteristic of incipient nano-optomechanical transduction. Associated photochromic changes are revealed by single-crystal optical-absorption microscopy. We show that the η^1^-OSO photoisomer that forms in **1** is metastable at 100 K with a 70% photoconversion fraction. A series of crystallographic data is also acquired once the crystal has been exposed to a temperature of 110 K, which is just above its metastable temperature. The rate of thermally induced reverse isomerization is slow enough at 110 K that we can crystallographically track the characteristic conversion of the η^1^-OSO photoisomer into a side-bound η^2^-(OS)O configuration; thus, we determine the structural mechanism of this transition. An additional series of crystallographic data on **1** is acquired once the crystal has been further exposed to an increase in temperature, 200 K, which we use to monitor the progressive reduction of its photoconversion fraction as **1** reverts to its S-bound η^1^-SO_2_ dark-state structure (Scheme 1c). We employ single-crystal Raman spectroscopy to corroborate these photoisomerization and thermally induced reverse isomerization processes.

## 2 Experimental Methods

Compound **1** was synthesized from *trans*-[Ru(SO_2_)(NH_3_)_4_Cl]Cl, which was synthesized according to a literature procedure.^52^ Five milligrams (16 μmol) of this precursor were dissolved in water (1.0 mL) to which 3-fluoropyridine (5 μL, 58 μmol) was added, and this induced a color change from orange to yellow. *p*-tosylic acid (200 μL, 2 M; > 98% purity, Sigma Aldrich) was then added dropwise to this solution. This produced a precipitate of orange platelike crystals after 2–4 h, which were isolated through vacuum filtration and washed three times with methanol.

Dark- and light-induced structures of **1** were characterized by photocrystallography^48−51^ using a three-circle Bruker diffractometer that was equipped with a monochromatic X-ray source (Mo Kα, λ = 0.71073 Å), an Apex CCD detector, and an Oxford Cryosystems open-flow N_2_ cryostream. A 505 nm light-emitting diode (LED) light source (ThorLabs M505F3, 1000 mA power output, 3.3 V forward voltage) was employed to photostimulate the largest face of the crystal for 2 h. The crystal was then illuminated for an additional 15 min at three rotated ϕ orientations, 90, 180, and 270° from this primary face, i.e., the crystal was photostimulated for 2 h, 45 min in total for each photocrystallography experiment. This light was switched off before acquiring data for each light-induced crystal structure. While the crystal was photoinduced exclusively at 100 K, it was either probed with X-ray diffraction at 100 K or exposed to a temperature increase of 110, 190, or 200 K before X-ray diffraction data were acquired. A single-crystal optical-absorption spectroscopy and microscopy setup was employed to determine the metal-to-ligand charge-transfer (MLCT) characteristics, thermal stability, and photochromic behavior of **1**. The 505 nm LED light provided an optical pump, while the 230 μm × 115 μm × 20 μm crystal of **1** was held at 100 K via an optical cryostat (Janis: ST-500-UC). The light excitation power measured at the 5×, 0.13 NA objective (Olympus, NeoSPlan) was typically 650 μW, giving an estimated 29.5 μW/mm^2^ illuminating the field of view. An inverted microscope (Olympus: IX71) coupled to a 300 mm focal length spectrograph (Princeton Instruments: Acton Series 2300i) and 1320 × 100 channel CCD camera (Princeton Instruments: PIXIS 100BR) was used to acquire spectra and micrographs of **1** at 100, 120, and 125 K (when optically pumped at 100 K). Single-crystal Raman spectroscopy on **1** was employed to characterize its molecular structure and thermal stability in more detail. Detailed experimental procedures for all of these *in situ* light-induced single-crystal characterization methods are given by Cole et al.^43^

## 3 Results and Discussion

### 3.1 Incipient Nano-optomechanical Transduction in **1** at 100 K

#### 3.1.1 Dark-State and Light-Induced Crystal Structures of **1**

**Figure 1:**
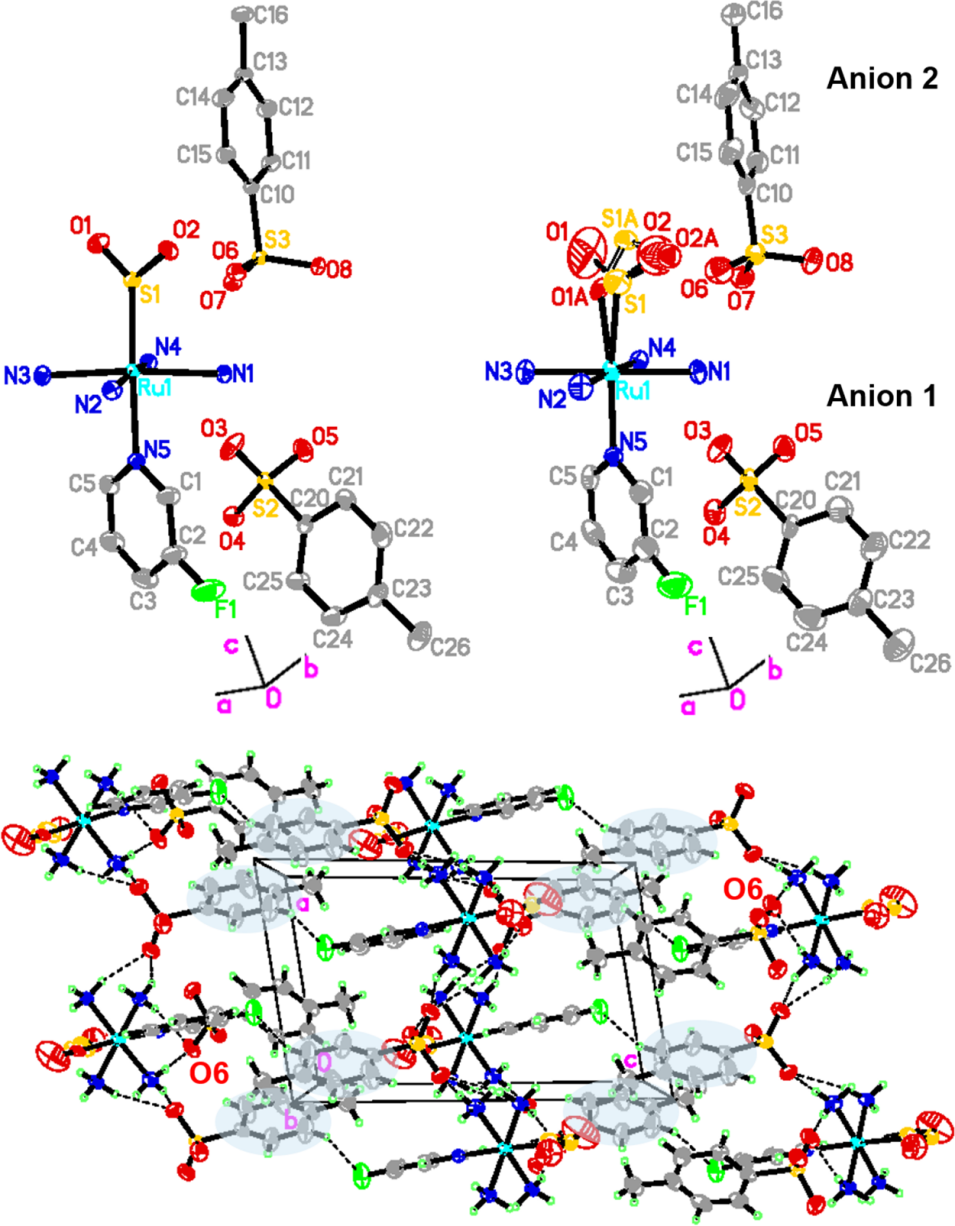
Crystallographic asymmetric units of the (top, left) dark- and (top, right) light-induced states of **1**. (bottom) Crystal packing diagram of **1** with the shading of arene rings that experience the effects of incipient nano-optomechanical transduction.

Crystallographic asymmetric units of the dark-state and light-induced crystal structures of **1** are displayed in Figure 1(top, left) and Figure 1(top, right), respectively. The dark-state structure is ordered, and all of its structural features appear to be regular, even the fluorine atom in the cation whose anisotropic displacement parameters (ADPs) are typical for a terminal chemical substituent of its sort of atomic mass. This is consistent with the dark-state crystal structures of its chloropyridine^38^ and bromopyridine^44^ analogues, all of which differ from its iodopyridine analogue,^46^ whose iodo atom is disordered; this has been attributed to the lower p*K*^a^ value of 3-iodopyridine and its consequentially weaker π-accepting and stronger σ-donating ability as a coordinative ligand to ruthenium, which makes it easier to undergo free rotation about its Ru-N_pyr_ bond (*cf.* p*K*^a^ values for 3-halopyridine ligands: 2.7 (F); 3.31 (Cl); 3.45 (Br); 3.5(I)). The light-induced crystal structure of **1** is similar to those of its chloro,^38^ bromo,^44^ and iodo^46^ analogues, with three notable differences: its η^1^-SO_2_ to η^1^-OSO photoconversion fraction; the level by which the ADP of its fluorine increases owing to photoisomerization; the extent of arene rotation in its tosylate anion that is involved in nano-optomechanical transduction. These differences are now detailed in turn.

η^1^-SO_2_ to η^1^-OSO photoisomerization is incomplete in **1**; its photoisomer forms with a 70.3(6)% occupancy. While this photoconversion fraction is not unusual in the wider set of [RuSO_2_] complexes, it contrasts markedly to the situation with its bromo and iodo analogues, which exhibit 100% photoconversion.^44,46^ It should be noted that this particular comparison was not extended to the chloro analogue, as its photocrystallography results were produced using a different (white) light source,^38^*cf.* a 505 nm LED source was used to study the other 3-halopyridine-based complexes.^44,46^

The ADP of the fluoro-substituent in **1** has *not* increased markedly relative to that of its dark-state structure, unlike the halogens in its three 3-halopyridine analogues;^38,44,46^ their ADPs are much larger in their light-induced crystal structures, especially in the case of the bromo- and iodo-based complexes.^44,46^ Indeed, the extent to which the ADPs of these halogens increase upon photoisomerization is deemed to be part of the nano-optomechanical transduction mechanism.

Only a small extent of arene rotation was observed in **1** within the tosylate anions that are engaged in nano-optomechanical transduction in the light-induced crystal structures of *trans*-3-halopyridine-based [RuSO_2_] complexes. The SO_2_-linkage photoisomerization process caused this arene ring to rotate by 39(4), 68(3), and 77(3)° in the chloro,^38^ bromo,^44^ and iodo-based^46^ complexes, respectively; in contrast, this rotation is too small to be quantified in the light-induced crystal structure of **1**; it is observable by comparing the ADPs of the carbon atoms in this ring, especially C21, C22, C24, and C25, either between those of the dark-state and light-induced crystal structures or comparing these ADPs against those of the other anion in the light-induced crystal structure (i.e., C11, C12, C14, and C15). For example, the ADPs of carbon atoms in the nonrotor ring of **1** (anion 2) are pretty isotropic when viewed edge-on in its light-induced crystal structure (Figure 1, top right) while the carbon atoms in the rotor ring of **1** (anion 1) can be seen to be distinctly anisotropic along the direction of the crystallographic *a* axis, as shown in Figure 1(bottom). The extent of this arene ring rotation in **1** is too small to partition the electron densities of their respective atomic contributions into separate structural components, and thus quantify its degree of rotation; however, it is clearly evidenced and caused by the SO_2_ photoisomerization in **1** whose light-induced structural changes are determined. Thus, we categorize this nano-optomechanical transduction in **1** as incipient.

The crystallographic unit-cell parameters of **1** are analogous to all three 3-halopyridine-based crystal structures in both their dark- and light-induced states (for details, see the Supporting Information). The crystal lattice of the light-induced crystal structure of **1** is shown in Figure 1(bottom), as viewed looking down the crystallographic *b* axis. The dark-state (η^1^-SO_2_) and light-induced η^1^-OSO configurations of the SO_2_ ligand display large ADPs therein, as one would expect. Furthermore, the oxygen atom in the η^1^-OSO photoisomer that protrudes from the cation (O2A) is directed toward the arene ring rotor (anion 1), as can be seen in the crystal packing diagram of **1** [Figure 1(bottom)]. In turn, anion 1 of **1** interacts with the fluorine atom of its cation via a C24-H24···F1 hydrogen bond (2.556(9) Å, 154.3(6)°; see the dashed line emanating from F in Figure 1(bottom)). This interaction would appear to stabilize the fluorine atom within the comparatively large volume in which it resides. Such stabilization could explain the lack of a significant increase in the ADP of the fluorine atom upon photoisomerization, in contrast to its three halogen-based analogous complexes.^38,44,46^ The larger and heavier atomic nature of the other halogens may also play an important role in this observed difference, as suggested by Cole et al.^46^ as well as help explain the difference in the η^1^-SO_2_ to η^1^-OSO photoconversion fractions that are observed across this halogen series. In the latter regard, the size and shape of the SO_2_ reaction cavity are also expected to be significant contributory factors in governing the level of such photoisomerization that is observed in [RuSO_2_] complexes.^41^

#### 3.1.2 Single-Crystal Optical Absorption Spectra of **1** as a Function of Photoisomerization Time

**Figure 2:**
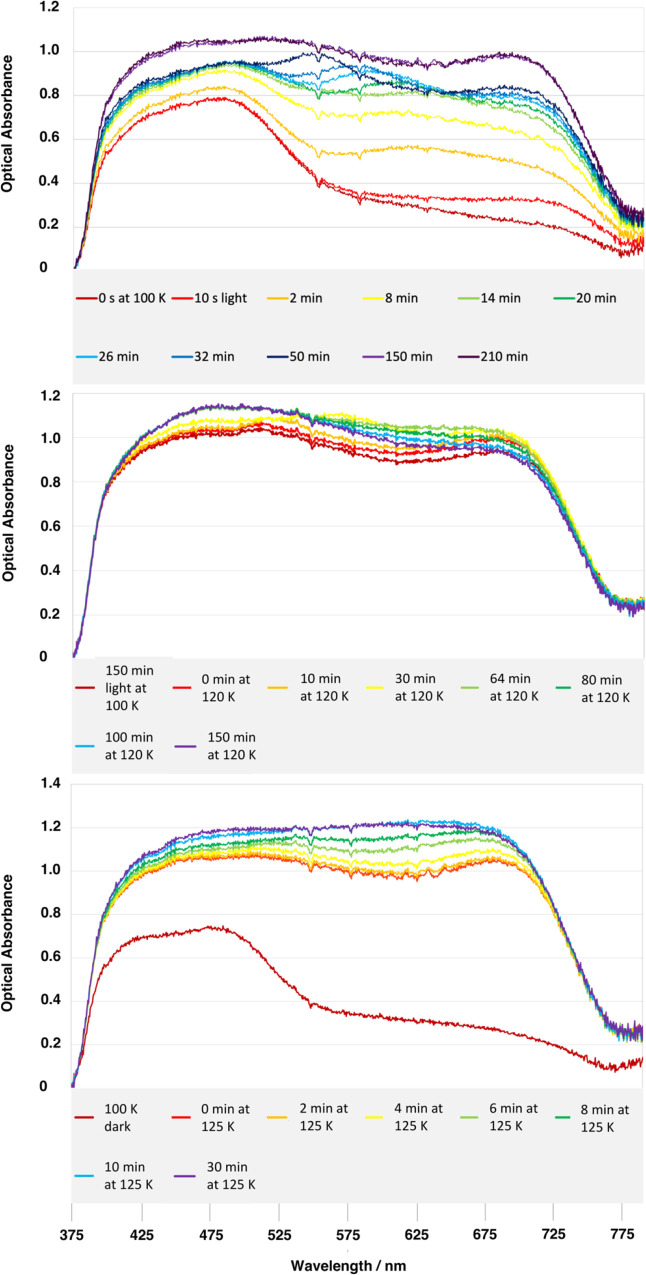
Selected single-crystal optical-absorption spectra of **1** as a function of (top) duration of 505 nm light exposure while the crystal was held at 100 K; (middle/bottom) elapsed time once the crystal has reached (middle) 120 K or (bottom) 125 K from 100 K, where it was induced by 505 nm light. For further details, see Figure S2.

Further evidence for the incipient nature of nano-optomechanical transduction in **1** at 100 K was observed via changes in its single-crystal optical-absorption spectral features as a function of light-exposure time (Figure 2, top; for the full range of spectra, see Figure S1). Thereby, an initial rise in optical absorption within the 600–750 nm region of its spectrum evolves into a substantial hypsochromic shift that progresses through to *ca*. 500 nm, where a peak in absorption is observed once **1** has been exposed to 90 min of 505 nm light, following which a fairly uniform panchromatic increase in single-crystal optical absorption is observed in **1** until light has been applied for *ca.* 130 min, whereby this absorption essentially becomes saturated, as shown by its minimal further increase up to a total light-exposure time of 240 min. This sequence of changes in the single-crystal optical-absorption spectra of **1** is likewise observed in its bromo^44^ and iodo^46^ analogues, although the exact wavelengths and optical-absorption values differ between these studies. Such a trend is similarly observed in the 3-methylpyridine analogue of these [RuSO_2_] complexes that also behave as a nano-optomechanical transducer.^45^ The distinguishing features in these sequences of optical-absorption changes are much more subtle in **1** but are nonetheless apparent. This corroborates the incipient nature of nano-optomechanical transduction that we categorized for **1** according to the single-crystal X-ray diffraction results.

These single-crystal optical absorption spectra were acquired alongside concerted single-crystal optical microscopy, whereby the photochromic changes in this crystal were imaged at each light-exposure time point. The resulting time sequences of images were cast into a movie (Supporting Information, Movie S1), which reveals a progressive color change in the crystal, from an orangy-yellow→greeny-gray→very dark gray hue, over a total duration of 4 h of 505 nm light application at 100 K. These color changes are akin to the photochromic changes that have been witnessed in the bromo (yellow→yellowy-green→gray)^44^ and iodo (yellow→greeny-gray→gray)^46^ analogues of **1** upon the progressive application of 505 nm light. The chloro analogue showed different color changes (orangy-red→green) in the study by Sylvester et al.,^38^ but this example is not truly comparable because a broad-band light was used for the photoisomerization process. It should also be borne in mind that the apparent hue and color intensity of each single crystal that was observed in the fluoro-, bromo-, and iodo-based studies will vary with the crystal thickness through which light must penetrate in order that it can be detected, remembering that light must penetrate through the entire crystal in each experiment because their single-crystal optical-microscopy images were all collected in transmission mode.

### 3.2 Thermally Induced Reverse Isomerization in **1**

#### 3.2.1 Single-Crystal Optical-Absorption Spectra of **1** Once Warmed from 100 to 120 or 125 K

This light-induced spectral profile of **1** undergoes a fairly uniform panchromatic rise in optical absorption once the temperature of its crystal is increased from 100 to 120 K (Figure 2, middle). Such changes are characteristic of an η^1^-OSO to η^2^-(OS)O thermal reverse isomerization process, as are observed in the bromo analogue of **1** at a similar temperature. This trend is likewise echoed when the crystal temperature of **1** is increased from 100 K to the slightly higher temperature of 125 K, although the rate of change is faster (see Figure 2, bottom). Indeed, this thermal reverse-isomerization process is deemed to operate in a thermodynamic fashion in [RuSO_2_] complexes once these crystals lie above their metastable temperature.^38−40,43−46^ Therefore, if one elevates a crystal of **1** to a temperature that is even closer to, but just above, its metastable temperature, one can monitor its thermal reverse-isomerization process over a much longer timeframe.

#### 3.2.2 Thermally Induced Reverse Isomerization in **1** Tracked by Time-Sequences of Single-Crystal X-Ray Diffraction Data

The temperature of the crystal of **1** that underwent the single-crystal X-ray diffraction experiment was increased to 110 K after its light-induced crystal structure characterization data were acquired at 100 K, following which a sequence of shorter sets of X-ray diffraction datasets (only phi scans) was collected repeatedly as a function of elapsed time that the crystal remained held at 110 K. This experiment enabled the η^1^-OSO to η^2^-(OS)O transition to be tracked structurally such that its mechanism could be unveiled via a sequence of crystal-structure determination. Subsequent η^2^-(OS)O to η^1^-SO_2_ transition that occurs at a higher temperature of around 190–200 K was also tracked in a similar fashion.

**Figure 3:**
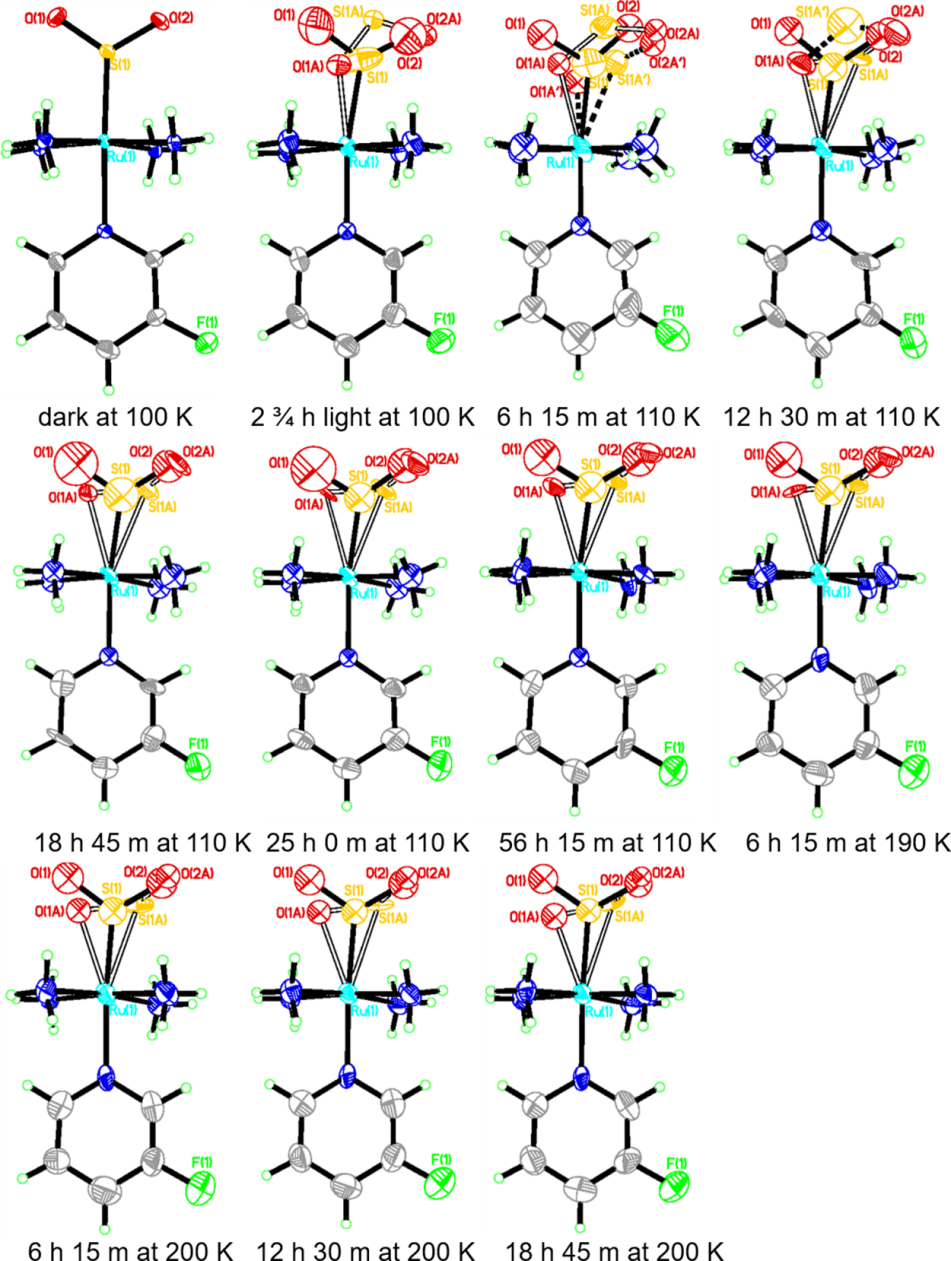
Cationic component of the crystal structure of **1** in its dark state at 100 K (top left) and its 505-nm-light-induced states at 100, 110, 190, and 200 K that evolves over the specified total time that they have been held at the subject temperature. The residual dark-state SO_2_ ligand contribution to the cationic structure of **1** is shown by solid bond lines; the dominant photoisomer (which evolves from the η^1^-OSO to η^2^-(OS)O configuration over time) is given in solid hollow bond lines; the more minor photoisomeric component is shown in open dashed bond lines.

The cationic crystal-structure components of **1** are shown in Figure 3 as a function of elapsed time at 110, 190, and 200 K, having exposed **1** to these temperatures progressively from the starting point of its 2 h 45 min light-induced crystal structure at 100 K. The cationic component of this 100 K light-induced crystal structure and that of its dark-state structure are also shown at the same orientation in the 110, 190, and 200 K plots for the purpose of comparison. Each cation is shown in an orientation that lies within the plane of the 3-fluoropyridine ligand.

##### 3.2.2.1 Bond-Geometry Changes Associated with SO_2_ Photoisomerization in **1** at 100 K

Light-induced configurational changes in the SO_2_-linkage isomer of **1** at 100 K are first discussed in terms of the effect of SO_2_ photoisomerization on its bond geometry and that of its coordinative environment; this helps lay the foundations for their structural analysis at elevated temperatures. The residual (29.7(6) %) S-bound η^1^-SO_2_ dark-state geometry in the light-induced crystal structure at 100 K is distorted along its S1-Ru1-N5 axis (170.6(3)°) relative to its 100% dark-state crystal structure (177.85(8)°). This distortion reflects its weakened coordination to the Ru ion owing to the formation of the 70.3(6)% O-bound η^1^-OSO photoisomer (*cf.* Ru1-S1 = 2.1135(9) Å (100% dark); 2.178(11) Å (after 2 h 45 min light)). The η^1^-OSO species coordinates well with the Ru ion (Ru1-O1A = 1.998(9) Å), as is evidenced via the adjoining bond length, O1A-S1A (1.499(8) Å), which is significantly longer than that of its SO_2_ counterpart, O2A-S1A (1.437(7) Å); the associated O1A-S1A-O2A bond angle is 117.7(18)°. This contrasts with the internal SO_2_ bond geometry of the dark-state structure, which displays uniform S-O bond lengths (S1-O1: 1.449(3) Å; S1-O2: 1.444(3) Å) and a slightly smaller O1-S1-O2 bond angle of 115.4(2)°.

The extent to which the η^1^-OSO photoisomer coordinates to the Ru ion can also be gauged by the angle, O1A-Ru1-N5, which is almost linear (173.0(2)°) at 100 K. The greater the tendency of this angle toward linearity, the stronger the coordination that is suggested. Interestingly, this angle is comparable to that of the aforementioned level of dark-state distortion in **1** at this temperature, as signified by the S1-Ru1-N5 angle (177.85(8)°). Thus, the η^1^-SO_2_ and η^1^-OSO configurations both appear to coordinate well to the Ru ion.

##### 3.2.2.2 Tracking the η^1^-OSO to η^2^-(OS)O Transition in **1** that Evolves at 110 K

The η^1^-OSO configuration is known to thermally decay into its η^2^-(OS)O photoisomer in [RuSO_2_] complexes. This work not only shows that **1** is consistent with the behavior of other [RuSO_2_] complexes, but also reveals the structural mechanism that is responsible for this η^1^-OSO to η^2^-(OS)O transition. The mechanism is depicted primarily across the first row of frames in Figure 3. This displays a time sequence of cationic crystal structures that evolve at 110 K, once the crystal temperature is increased to 110 K from 100 K. Therein, a portion of the photoconverted η^1^-OSO species initially shifts into a pseudo-η^1^/η^2^-OSO intermediate structural configuration after being held for 6 h 15 min at 110 K. Thereby, the S atom in the η^1^-OSO configuration has started to pivot in a fashion that places it sufficiently close to the Ru ion that it can coordinate with this metal. The O atom that is already coordinated to this Ru ion makes room for this (S,O)-side-bound coordination to take hold. Indeed, the corresponding Ru-O1A-S1A angle becomes less obtuse in order to accommodate these structural perturbations while the (S,O)-side-bound coordination grows and its initially distorted geometry settles into the η^2^-OSO photoisomeric form that dominates the SO_2_ structure by 12 h 30 min at 110 K, with a photoconversion fraction of 54(2)% (see Table 1). The configuration of the SO_2_ ligand continues to shift until its photoisomeric component evolves into the full η^2^-(OS)O geometry, by which point the η^1^-OSO photoisomer fully converts into this species. This conversion appears to have occurred by at least 18 h 45 min after the crystal temperature of **1** had been increased from 100 K and held at 110 K, judging from the SO_2_ isomeric fractions of **1** that were refined in the crystal structure at this time (η^1^-SO_2_: η^2^-(OS)O = 34(3): 66(3); *cf.*Table 1). This ratio is within the experimental error of the 29.7(6): 70.3(6) η^1^-SO_2_: η^1^-OSO dark-state: light-induced state proportioning that was observed in the crystal of **1** upon application of light at 100 K. Thus, it would appear that the residual dark-state η^1^-SO_2_ species in **1** remains at 30% throughout the η^1^-OSO to η^2^-(OS)O transition, while the η^1^-OSO photoisomer wholly migrates into its other photoisomer. Nonetheless, a temporary modulation in the internal bond geometry of the dark-state η^1^-SO_2_ species occurs during this η^1^-OSO to η^2^-(OS)O transition, presumably as a response to this structural perturbation. This modulation is best witnessed via changes in the O1-S1-O2 angle that are observed across the full timed sequence of structure determinations, as shown in Table 1. The O1A-S1A-O2A bond angles of the dominant photoisomeric configuration undergo a similarly ephemeral modulation as it evolves from the η^1^-OSO to η^2^-(OS)O isomer, as is likewise recorded in Table 1. A significant change in *trans* influence is also witnessed during this transition via the temporary reduction in the Ru1-N5 bond length; this stands to reason, given that the SO_2_-ligand coordination will become turbulent as its bonding mode changes from an O-bound to side-bound (S,O)-configuration.

**Table 1:** Sequence of Relevant Changes in the Bond Geometry and Photoconversion Fractions of **1** That Are Associated with Its Two Thermally Induced Reverse-Isomerization Processes^a^

hours of light at *T*	η^1^SO_2_:η^2^(OS)O:η^1^-OSO	O1A-S1A-O2A	O1-S1-O2	S1-Ru1-N5	Ru1-O1A	Ru1-S1A	Ru1-S1	S1A-O1A	S1A-O2A	S1-O1	S1-O2	Ru1-N5	R1 (I > 2σ(I))%
2 h 45 min at 100 K	29.7(6):0:70.3(6)	115.0(4)	117.7(18)	170.6(3)	1.998(9)		2.178(11)	1.499(8)	1.437(7)	1.389(10)	1.407(10)	2.066(4)	7.05
6 h 15 min at 110 K	21(3):28(3):42(3)	126.9(17)	107(6)	170.6(12)	2.23(4)		2.18(4)	1.504(10)	1.439(10)	1.402(11)	1.402(11)	1.994(13)	11.08
12 h 30 min at 110 K	24(2):54(2):21(3)	123.6(14)	107(5)	171.4(10)	2.16(2)	2.433(12)	2.00(3)	1.447(10)	1.415(10)	1.404(10)	1.401(11)	2.058(12)	9.42
18 h 45 min at 110 K	34(3):66(3):0	120.4(14)	114(4)	171.4(9)	2.19(2)	2.432(9)	2.07(3)	1.505(18)	1.413(10)	1.397(10)	1.400(10)	2.055(10)	8.57
25 h 0 min at 110 K	31(3):69(3):0	116.6(16)	113(6)	171.0(8)	2.24(2)	2.428(9)	2.02(3)	1.50(2)	1.419(10)	1.400(10)	1.401(10)	2.066(12)	8.99
56 h 15 min at 110 K	31(2):69(2):0	121.9(8)	116(3)	170.6(5)	2.147(12)	2.407(5)	2.050(15)	1.509(10)	1.393(8)	1.383(10)	1.404(10)	2.076(7)	5.61
6 h 15 min at 190 K	31(2):69(2):0	115.7(7)	116(2)	171.6(4)	2.121(11)	2.407(4)	2.040(12)	1.516(9)	1.428(12)	1.34(3)	1.39(3)	2.059(6)	4.63
6 h 15 min at 200 K	48(2):52(2):0	116.8(9)	113.4(13)	173.1(3)	2.183(16)	2.383(6)	2.028(9)	1.494(15)	1.435(15)	1.398(18)	1.390(18)	2.084(7)	5.48
12 h 30 min at 200 K	70(2):30(2):0	113.7(15)	115.6(8)	175.06(18)	2.21(3)	2.438(9)	2.071(5)	1.55(2)	1.43(2)	1.406(12)	1.411(11)	2.100(6)	5.35
18 h 45 min at 200 K	82(1):18(1):0	116(2)	116.3(6)	175.95(15)	2.12(4)	2.481(14)	2.093(3)	1.54(3)	1.44(3)	1.395(9)	1.423(8)	2.119(6)	5.36

a Bond lengths and angles are given in Angstroms (Å) and degrees (°).

The η^2^-(OS)O isomer had stabilized at least by the time that the crystal of **1** had remained at 110 K for 56 h 15 min, judging from the stable 31(2):69(2) photoconversion fraction of the associated crystal-structure determination and its good-quality statistical figure-of-merit, R1 (I > 2σ(I)), whose markedly lower value compared with other 110 K refinements (see Table 1) indicates a more ordered and stable structure.

##### 3.2.2.3 Tracking the η^2^-(OS)O to η^1^-SO_2_ Transition in **1** That Evolves at 200 K

Having reached thermal stability at 110 K, the temperature of the crystal of **1** was then increased, first to 190 K and then to 200 K, in order to structurally track the η^2^-(OS)O to η^1^-SO_2_ transition as **1** returns to its dark-state configuration. No thermally induced photoisomeric decay was observed in the crystal structure of **1** that was determined at 190 K, judging from its refined η^1^-SO_2_: η^2^-(OS)O ratio, which was stationary relative to that of the stabilized 110 K structure (see Table 1). The crystal was therefore exposed to a slightly higher temperature of 200 K, whereupon its η^1^-SO_2_: η^2^-(OS)O ratio was monitored via three successive crystal-structure determinations from data that were acquired over a total time period of 18 h 45 min from the point at which the crystal had reached 200 K. Table 1 reveals that the η^1^-SO_2_: η^2^-(OS)O ratio of **1** depletes progressively while being held at 200 K, as the crystal reverts to its dark-state configuration. The *trans* influence decreases over the course of this transition, as is evidenced by the progressive increase in the Ru1-N5 bond length seen in **1** during this time period (Table 1). The S1-Ru1-N5 angle also increases during this transition, becoming almost entirely linear (175.96(15)°) by the end of this time period, at which point the η^1^-SO_2_ dark-state structure of **1** has nearly fully recovered. The S1-O1, S1-O2, and Ru1-S1 bonds also appear to lengthen during this time (see Table 1), although these “observations” will at least partially be a manifestation of the progressive reduction in the structural disorder of the SO_2_ ligand, which causes an artificial foreshortening of these bond lengths. Nonetheless, the structural disorder in SO_2_ at 200 K is not as substantial as it is at 110 K, judging from the plots of the cationic part of the structure of **1** at these two temperatures (Figure 3) and the steady and good statistical figure-of-merit, R1(I > 2σ(I)), that is associated with the crystal-structure model refinements against data that were acquired at 200 K.

### 3.3 Thermally Induced Changes in the Anions of **1**

**Figure 4:**
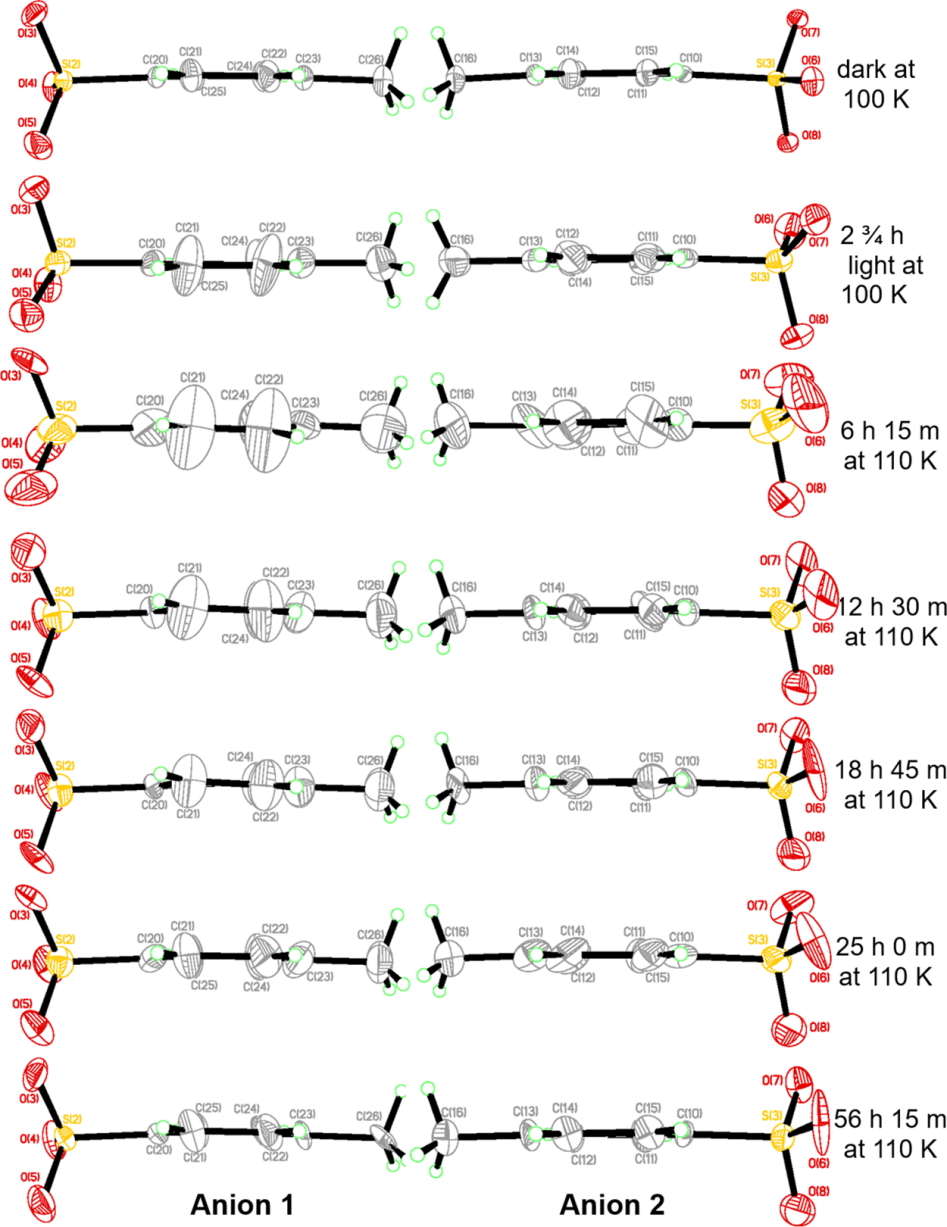
Two anionic components of the crystal structure of **1** in their dark state at 100 K (top) and their 505-nm-light-induced states at 110 K, which evolve over the specified total time that they have been held at the subject temperature. The ADPs in anion **1** display the effects of incipient nano-optomechanical transduction while those in anion 2 vary according to the knock-on effects of the progressive transition of the SO_2_ ligand from the η^1^-OSO to η^2^-(OS)O configuration.

**Figure 5:**
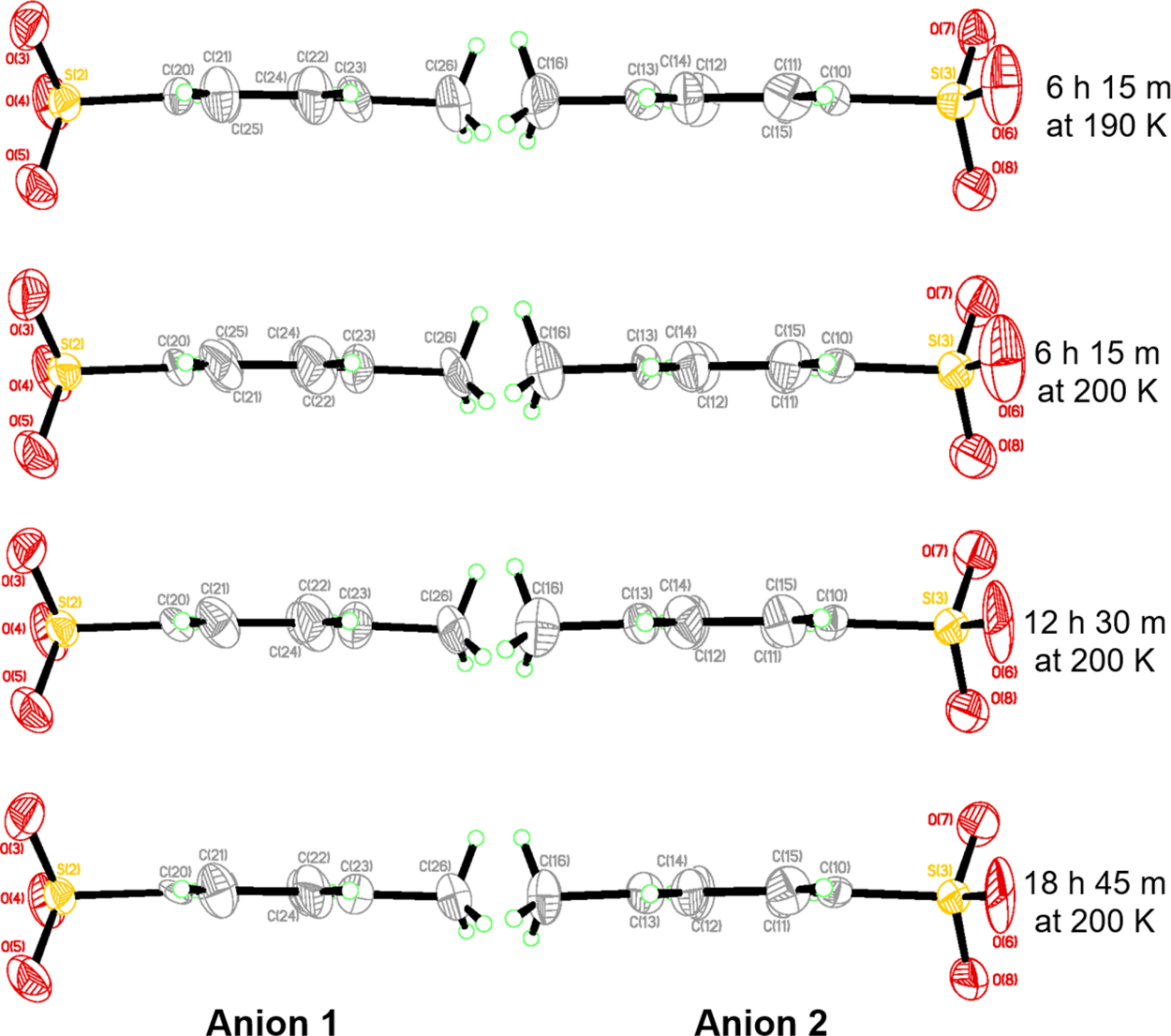
Two anionic components of the crystal structure of **1** in their 505 nm light-induced states at 190 and 200 K, displayed at the specified total time that they have been held at the latter temperature. The ADPs in both anions appear to be quite stable despite the η^2^-(OS)O to η^1^-SO_2_ transition that occurs concurrently within their neighboring crystal-lattice environment.

The structural components of both tosylate anions in **1** are displayed as the temperature is increased from 100 to 110 K (Figure 4), and then to 190 and 200 K (Figure 5). The arene rings in these anions are therein presented perpendicular to the page, so as to elucidate the incipient nature of the nano-optomechanical transduction in anion 1 of **1** via their ADPs that evolve as a function of elapsed time at 110 K. The ADPs of the nonrotor (anion 2 in **1**) are shown aside those of anion 1.

The ADPs of C21, C22, C24, and C25 in anion 1 become distinctly elongated in the direction perpendicular to its arene ring at 100 K, once 2 h 45 min of 505 nm light is applied to **1**. This contrasts with the essentially spherical form of the ADPs of the analogous carbon atoms in anion 2 of **1** (C11, C12, C14, and C15) under these same light-induced conditions although their magnitude has slightly increased relative to the dark-state structure at 100 K, which stands to reason because the light imposes a structural perturbation in **1** that will have a knock-on effect on the ADPs of **1**. The markedly increasing ADPs in anion 1 of **1** are a manifestation of the incipient nature of the nano-optomechanical transduction (*vide supra*).

Once the crystal temperature of **1** is increased from 100 to 110 K, all of its ADPs increase substantially, far more than one would expect purely on account of the slight thermal elevation. Indeed, the pronounced elongation that was seen in the aforementioned ADPs of anion 1 in **1** at 100 K becomes especially accentuated by the time that the crystal has been held at 110 K for 6 h 15 min. This reflects the onset of thermal instability in the incipient nano-optomechanical transducer that occurs as the η^1^-OSO photoisomer, which induces the transduction, starts to transition to the η^2^-(OS)O configuration. A substantial level of structural perturbation is exhibited in the region of the SO_2_ reaction cavity as a result of this η^1^-OSO to η^2^-(OS)O transition. The gradual loss of the η^1^-OSO photoisomer as the crystal of **1** continues to be held at 110 K diminishes the ability of **1** to behave as a nano-optomechanical transducer; thus, the relevant ADPs of anion 1 in **1** reduce in size over time until they become essentially the same as those of the ADPs at 100 K once the η^2^-(OS)O isomer becomes thermally stable after being held for 56 h 15 min at 110 K. In addition, the shapes of ADPs in anion 1 and anion 2 become essentially the same by this time. Thereafter, the size and shape of these ADPs seem to remain the same, save for changes that one would naturally expect with increasing thermal effects as the temperature is increased to 190 and 200 K (see Figure 5). All effects of nano-optomechanical transduction have long disappeared once the crystal of **1** reached either of these two temperatures.

ADPs of other atoms in both anions of **1** appear to increase at the level that one would expect for thermal effects, except for when the crystal is held at 110 K where they are greater, owing to the depletion of nano-optomechanical transduction at this temperature. The ADPs of the SO_3_ groups in both anions of **1** display a degree of liberation at 110 K and above, although liberation is particularly pronounced in one oxygen: O(6) in the SO_3_ group of anion 2 in **1**, whose ADPs become large and markedly elongated. Figure 1 provides a rationale for this exception via its display of bifurcated nonbonded contacts that involve O(6) with hydrogens from two of the ammine ligands, for example, N(1)-H(2 N1)···O(6): 2.109(6) Å, 157.1(9)°; N(4)-H(1 N4)···O(6): 2.140(6) Å, 150.5(9)° after 12 h 30 min at 110 K; these will exhibit the effect of pulling O(6) in two opposing directions, which will thus constrain the shape of its ADP.

### 3.4 Corroboratory Multitemperature Single-Crystal Raman Spectra of **1**

**Figure 6:**
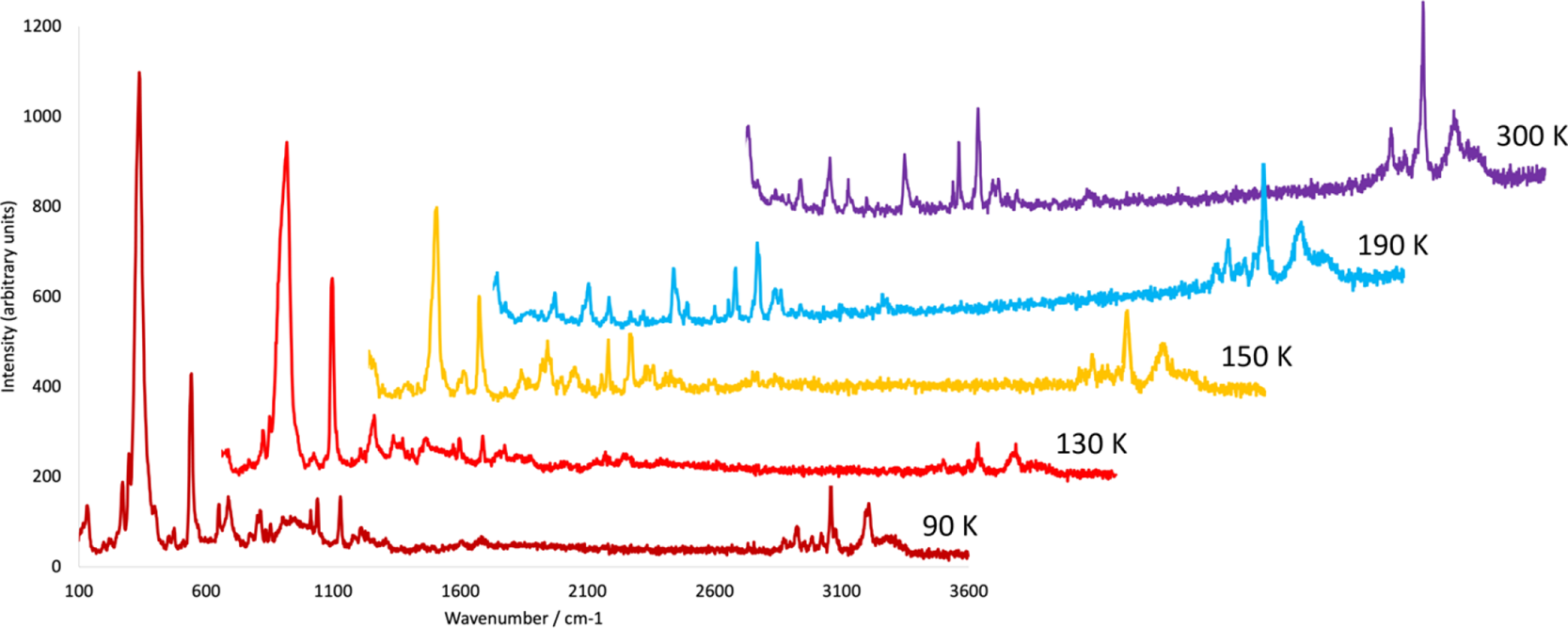
Single-crystal Raman spectra of **1** measured in its light-induced state as a function of increasing temperature, from 90 to 300 K, using the 514.5 nm laser light of the Raman spectrometer to pump and probe the sample. Results are displayed from only a limited range of the full frequency coverage of these spectra, for the sake of clarity; the full range of results are given in the Supporting Information (Figure S2).

The SO_2_ photoisomerization and thermally induced reverse-isomerization processes in **1** were also tracked by single-crystal vibrational spectroscopy, whereby the incident Raman light (514.5 nm) was used to pump and probe the crystal across the temperature range, 90–300 K. The salient Raman features that were detected are shown in Figure 6. Thereby, three peaks are significant at 90 K: the largest peak at 340 cm^–1^, which represents a Ru–OSO vibrational stretch;^44^ the more modest peak at 540 cm^–1^, which is indicative of an SO_2_ deformation mode of the η^1^-OSO isomer;^44,53^ and the weakest peak at 1130 cm^–1^, which is reminiscent of an η^1^-SO_2_ symmetric stretching mode.^43,44,53^

As the temperature of the crystal of **1** is elevated, distinct spectral changes first occur at 130 K: the peak at 340 cm^–1^ shifts to a more intense and a much broader peak that is centered at 360 cm^–1^, the latter being indicative of a Ru-(OS)O vibrational stretch;^43,44^^,54^ the peak at 540 cm^–1^ also becomes stronger at this temperature, where the η^2^-(OS)O isomer contributes more significantly to the deformation mode of SO_2_.^44^ These spectral shifts naturally reflect changes in vibrational modes that one would expect to be associated with the η^1^-OSO to η^2^-(OS)O transition.

These two vibrational modes start to decay as the temperature is increased further, until they appear negligible by *ca.* 170 K. The onset of other Raman peaks occurs as the temperature continues to increase from 170 to 230 K: a weak peak at 350 cm^–1^ forms, which is the characteristic of the Ru-S vibrational stretching mode;^43,44,54^ another weak peak appears at 550 cm^–1^, which is reminiscent of the deformation mode of SO_2_ for the η^1^-SO_2_ isomer.^44^ A significant broadening and increase in the intensity of the peak centered at 1130 cm^–1^ are also observed in this temperature range, which is due to the increasing population of the η^1^-SO_2_ symmetric stretching mode^43,44,53^ as **1** reverts to its dark-state crystal structure.

An increase in the intensity of several weak peaks that are characteristic of Ru-N_amine_ bending modes (480–490 cm^–1^)^53^ and amine rocking modes (815 cm^–1^)^52^ also becomes more apparent by *ca.* 230 K (see Figure 6), while vibrational modes that are associated with amine stretching modes are also present at 3200–3350 cm^–1^ (for details, see the Supporting Information).^52^

Overall, these Raman results corroborate those from the single-crystal optical-absorption spectroscopy and X-ray diffraction experiments in characterizing SO_2_ photoisomerization and the two thermally induced SO_2_ reverse-isomerization processes. On a practical note, it is important to note that the rate at which the temperature is increased in these single-crystal Raman-spectroscopy measurements is faster than that of those of the other two metrologies. Given the thermodynamic nature of SO_2_ reverse isomerization in [RuSO_2_] complexes, this means that the temperatures that are associated with the η^1^-OSO to η^2^-(OS)O transition and the η^2^-(OS)O to η^1^-SO_2_ transition will slightly differ between results from these different metrologies. However, these transitions consistently range from 110 to 130 K and from 170 to 230 K.

## 4 Conclusions

Our discovery of **1** completes the series of [Ru(SO_2_)(NH_3_)_4_(3-fluoropyridine)]tosylate_2_ complexes, all of which behave as single-crystal optical actuators. This 3-fluoropyridine specification has been structurally and optically characterized in its dark state and light-induced state, which result from SO_2_-linkage photoisomerization. Specialized single-crystal metrologies have been used exclusively to characterize **1** because it only exists in this form and therein presents its properties of interest. *In situ* single-crystal X-ray diffraction (so-called photocrystallography),^48−51^ concerted single-crystal optical-absorption spectroscopy and microscopy, and single-crystal Raman spectroscopy are employed for this purpose.^43^ Results have shown that **1** exhibits incipient nano-optomechanical transduction, whereby SO_2_-linkage photoisomerization in its ruthenium-based cation causes the arene ring in one of its tosylate anions to rotate by such a modest amount that the rotor can be observed structurally but not fully resolved. This contrasts with the 3-halopyridine analogues of **1** that display much greater extents of nano-optomechanical transduction such that arene ring rotation therein can be fully distinguished and quantified.

SO_2_-linkage photoisomerization has been found to proceed in **1** via an η^1^-SO_2_ to η^1^-OSO transition which is 70% complete, as determined via photocrystallography. Its light-induced crystal structure also revealed the incipient nature of nano-optomechanical transduction in **1**. Single-crystal optical-absorption spectral profiles of **1** corroborated this incipient classification. They also showed that the η^1^-OSO photoisomer in **1** decays thermally into an η^2^-(OS)O isomer when the temperature of the crystal increases from 100 to 110–125 K. Multiple-temperature single-crystal Raman spectroscopy was able to corroborate this finding.

We have captured the structural mechanism of this thermally induced SO_2_ reverse-isomerization process via a series of multitemperature single-crystal X-ray diffraction experiments. This study managed to structurally track two isomeric transitions in **1** that occur within the temperature range, 100–200 K: the η^1^-OSO to η^2^-(OS)O structural isomerization and an η^2^-(OS)O to η^1^-SO_2_ configurational change that returns **1** to its dark-state structure. While other structural studies have shown that these two isomeric transitions exist in certain [RuSO_2_] complexes;^38,39,43,44^ this study on **1** is the first to track the structural evolution of the SO_2_ species through these two thermally induced reverse-isomerization processes via a time sequence of fully refined structural models that were realized via crystallographic data that have been acquired slow enough to structurally monitor its operational mechanism.

We have found that the η^1^-OSO photoisomer thermally converts to its η^2^-(OS)O isomer via a mechanism that is reminiscent of coordination isomerism. Thereby, the η^1^-OSO configuration starts to pivot about its sulfur atom in a fashion that places it sufficiently close to the Ru ion that it coordinates with the metal; meanwhile, its bound oxygen atom remains coordinated to the Ru core, although in a weakened state owing to its newfound bonding competition with the sulfur atom. This (S,O)-side-bound coordinative form of the SO_2_-linkage photoisomer is initially distorted, but it gradually settles into a clean η^2^-(OS)O configuration while being held at 110 K over the course of a 56 h 15 min period. This η^2^-(OS)O isomer thermally reverts back to its η^1^-SO_2_ dark-state isomeric form over a period of more than 19 h while being at 200 K, as this (S,O)-side-bound SO_2_ photoisomer progressively converts back into its S-bound SO_2_ dark state.

The elucidation of these structural mechanisms is helpful to guide future work in the molecular design of nano-optomechanical transducers within this family of [RuSO_2_] complexes. The discovery and crystal-structure determination of **1** in its dark- and light-induced states also complete the synthetic and structural set of [Ru(SO_2_)(NH_3_)_4_(3-halopyridine)]tosylate_2_ compounds in this [RuSO_2_] family of complexes. The resulting ability to compare their structures across this series of halogen-containing substituted variations is also helpful to guide the future molecular design of these single-crystal optical actuators.

## Supporting Information Available

Material-characterization details for **1**; crystallographic information files of the dark- and light-induced state structure of **1** at 100 K as well as the light-induced structure of **1** once it had been raised from 100 to either 110, 190, or 200 K and held at that temperature for a specified time (PDF)
Photochromism changes in a single crystal of **1** observed as a function of light-exposure time when viewed by single-crystal optical-absorption microscopy (MP4)
X-ray crystallographic information (CIF)
X-ray crystallographic information (CIF)
X-ray crystallographic information (CIF)
X-ray crystallographic information (CIF)
X-ray crystallographic information (CIF)
X-ray crystallographic information (CIF)
X-ray crystallographic information (CIF)
X-ray crystallographic information (CIF)
X-ray crystallographic information (CIF)
X-ray crystallographic information (CIF)
X-ray crystallographic information (CIF)

## Supplementary Material

jp2c00170_si_002.mp4

jp2c00170_si_003.cif

jp2c00170_si_004.cif

jp2c00170_si_005.cif

jp2c00170_si_006.cif

jp2c00170_si_007.cif

jp2c00170_si_008.cif

jp2c00170_si_009.cif

jp2c00170_si_010.cif

jp2c00170_si_011.cif

jp2c00170_si_012.cif

jp2c00170_si_013.cif
