# Supplementary material for: Structural Capture of η1-OSO to η2-(OS)O Coordination Isomerism in a New Ruthenium-Based SO2-Linkage Photoisomer That Exhibits Single-Crystal Optical Actuation
Source: J Phys Chem C Nanomater Interfaces. 2022 Mar 29;126(13):6047–59. doi: 10.1021/acs.jpcc.2c00170 (PMC9098168; doi:10.1021/acs.jpcc.2c00170)
Supplement: Supplementary file 1 — jp2c00170_si_001.pdf [file jp2c00170_si_001.pdf]

Supporting Information for:

Structural Capture of η^1-OSO to η^2-(OS)O Coordination Isomerism in a New Ruthenium-based SO_2 Linkage Photoisomer that Exhibits Single-crystal Optical Actuation

Jacqueline M. Cole[1,2,3*], David J. Gosztola[3], Jose de J. Velazquez-Garcia[1]

[1]Cavendish Laboratory, Department of Physics, University of Cambridge, J. J. Thomson Avenue, Cambridge, CB3 0HE, UK.

[2]ISIS Neutron and Muon Source, STFC Rutherford Appleton Laboratory, Harwell Science and Innovation Campus, Didcot, OX11 0QX, UK.

[3]Center for Nanoscale Materials, Argonne National Laboratory, 9700 S Cass Avenue, Lemont, IL 60439, United States.

*Correspondence to: jmc61@cam.ac.uk

Table of Contents

S1 – Single-crystal optical absorption spectra of **1**.........S2

S2 – Single-crystal Raman Spectra of **1** showing the full frequency range measured.........S3

S3 – Movie of the progressive photochromic changes observed in a single-crystal of **1** as a function of light exposure time at 100 K ....(*separate file*)

S4 – Crystallographic information file for the light-induced state of **1** refined against data collected acquired at 100 K ....(*separate files*)

S5 – Crystallographic information file for the light-induced state of **1** refined against data collected at 110 K, 190 K or 200 K having photo-induced the crystal at 100 K and warmed up to the data acquisition temperature ....(*separate file*)

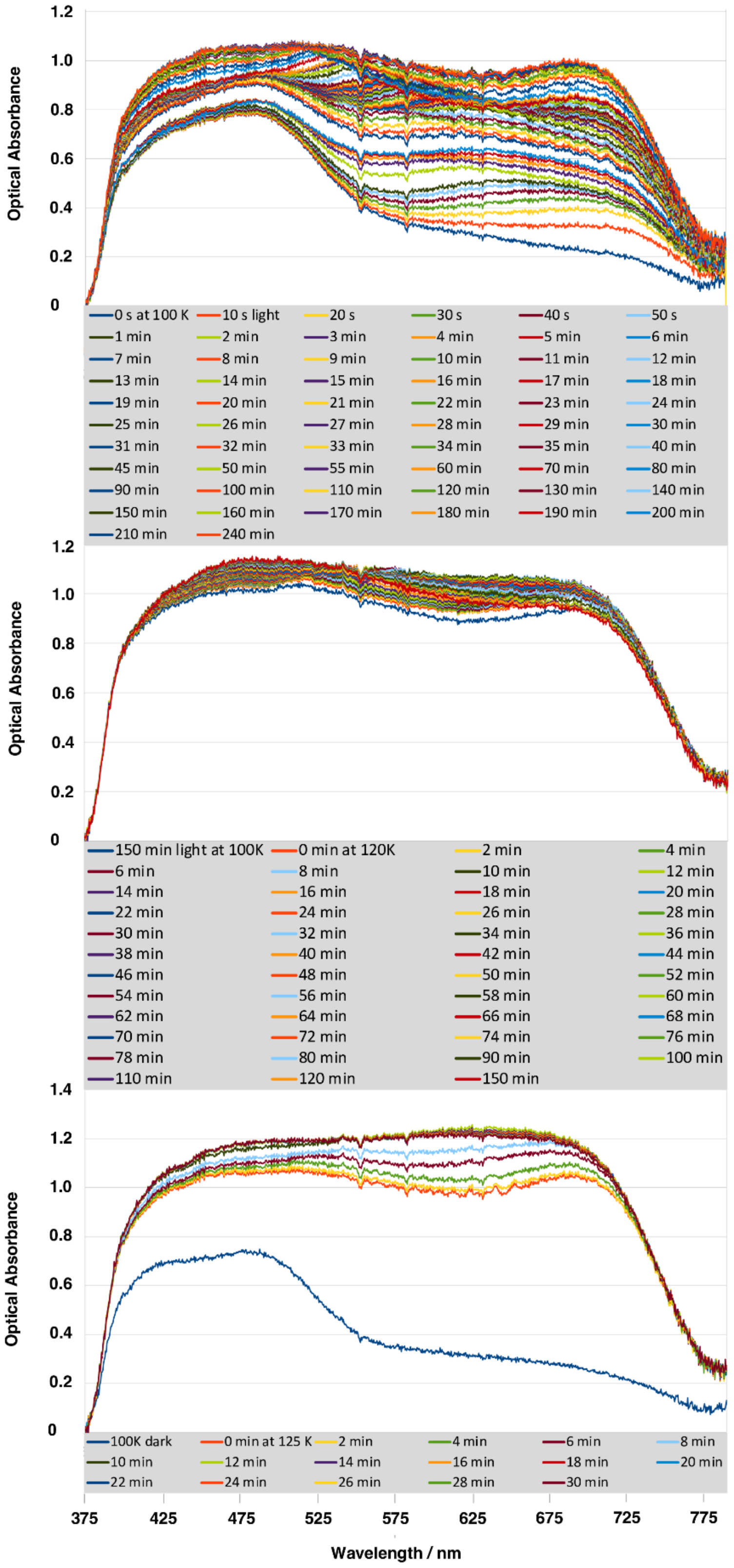

Figure S1: Single-crystal optical absorption spectra of **1** as a function of (top) duration of 505 nm light exposure while the crystal was held at 100 K; (middle/bottom) elapsed time once the crystal has reached (middle) 120 K or (bottom) 125 K from 100 K where it was induced by 505 nm light.

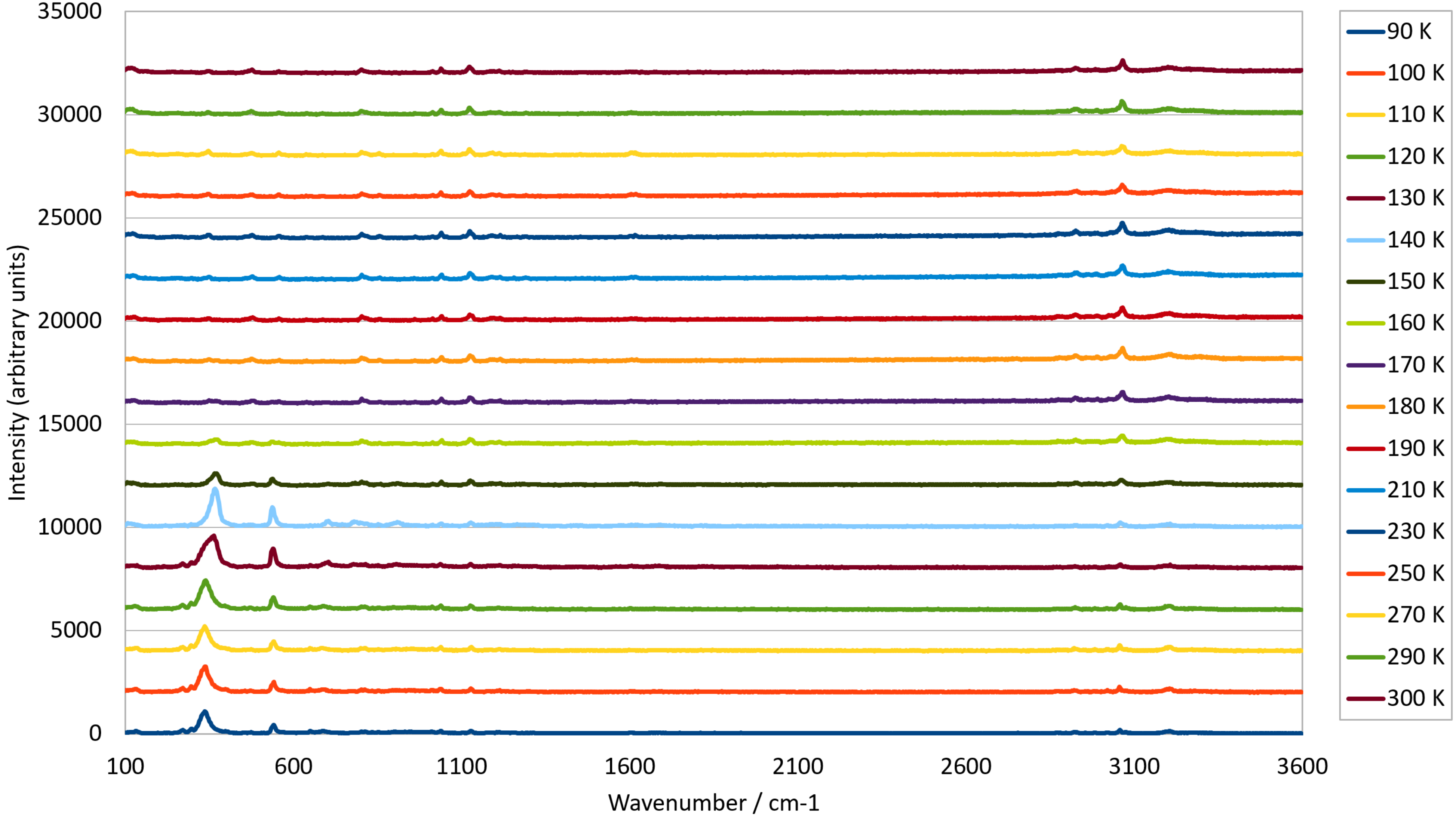

Figure S2 – Multi-temperature single-crystal Raman spectra of **1** showing the full frequency range measured.
